# Pathogenic free-living amoebic encephalitis from 48 cases in China: A systematic review

**DOI:** 10.3389/fneur.2023.1100785

**Published:** 2023-02-09

**Authors:** Xiang-Ting Chen, Qian Zhang, Si-Yuan Wen, Fei-Fei Chen, Chang-Qing Zhou

**Affiliations:** Department of Neurology, Bishan Hospital of Chongqing Medical University, Chongqing, China

**Keywords:** free-living amoebae (FLA), primary amoebic meningoencephalitis (PAM), granulomatous amoebic encephalitis (GAE), balamuthia amoebic encephalitis (BAE), *Naegleria fowleri*, *Acanthamoeba* spp., *Balamuthia mandrillaris*

## Abstract

**Background:**

Free-living amoebae (FLA) including *Naegleria fowleri, Acanthamoeba* spp., and *Balamuthia mandrillaris* can become pathogenic and cause severe cerebral infections, named primary amoebic meningoencephalitis (PAM), granulomatous amoebic encephalitis (GAE), and balamuthia amoebic encephalitis (BAE), respectively. FLA encephalitis has been reported across China, but the clinical data descriptions and analytical results of these different reports vary widely. Currently, no consensus treatment has been established. We conduct a systematic review to evaluate the exposure location, clinical symptoms, diagnosis, treatment, and prognosis of three FLA encephalitis and aim to reveal the differences between three FLA encephalitis in China.

**Methods:**

We used MEDLINE (PubMed interface), EMBASE, China National Knowledge Infrastructure (CNKI), Wanfang database, and China Biology Medicine disc (CBMdisc) databases for literatures published and manually retrieve the hospital records of our hospital. The search time was up to August 30, 2022, with no language restrictions.

**Results:**

After excluding possible duplicate cases, a total of 48 patients of three FLA encephalitis were collected. One from the medical records of our hospital and 47 patients from 31 different studies. There were 11 patients of PAM, 10 patients of GAE, and 27 patients of BAE. The onset of PAM is mostly acute or subacute, and the clinical symptoms are acute and fulminant hemorrhagic meningoencephalitis. Most patients with GAE and BAE have an insidious onset and a chronic course. A total of 21 BAE patients (77.8%) had skin lesions before onset of symptoms. Additionally, 37 cases (77.1%) were diagnosed with FLA encephalitis before death. And there were 4 of PAM, 2 of GAE, and 10 of BAE diagnosed using next generation sequencing. No single agent can be proposed as the ideal therapy by itself. Only 6 cases were successfully treated.

**Conclusions:**

This review provides an overview of the available data and studies of FLA encephalitis in China and identify some potential differences. FLA encephalitis is a rare but pathogenic infection, and physicians should early identify this encephalitis to improve survival.

## 1. Introduction

Free-living amoebae (FLA) are unicellular eukaryotes that can develop as free-living organisms in the environment or as parasites in hosts. *N. fowleri* (*Naegleria fowleri*), *Acanthamoeba* spp., and *B. mandrillaris* (*Balamuthia mandrillaris*) represent amphizoic amoebas that exhibit pathogenic and free-living species (1). They can reach the central nervous system (CNS) by hematogenous spread from a primary site in wounded skin or upper respiratory tract *via* nasal passage, which can cause serious cerebral infections, named primary amoebic meningoencephalitis (PAM), granulomatous amoebic encephalitis (GAE), and balamuthia amoebic encephalitis (BAE) respectively (2).

It is not entirely clear why different FLAs cause different types of encephalitis. From a phylogenetic point of view, *Acanthamoeba* spp. and *B. mandrillaris* are more closely related. Both of these pathogens have two developmental stages: the trophozoite as a nutrition feeding form and the cyst as a resting form. *N. fowleri* presents an additional flagellated stage. In the presence of water, the trophozoite transitions to this temporary motile stage and allows the amoebae to find other favorable environmental niches. Likewise, recent studies (3, 4) have shown that the immune response to the three FLAs is different. *Acanthamoeba* spp. and *B. mandrillaris* primarily involve macrophages and T cells, and induce the formation of granulomas. In contrast, *N. fowleri* elicits an acute inflammatory response that primarily involves neutrophils and macrophages, the production of pro-inflammatory cytokines, and severe tissue damage (5).

The clinical symptoms of these three cerebral infections are similar, but brain inflammation and disease course are different. In the early stage, general symptoms such as headache and fever may appear. And according to the distribution and severity of the lesion, neurological symptoms such as confusion, hemiparesis and seizures may appear. Currently, pathogenic FLA encephalitis is an infectious disease with a high mortality rate due to the small number of cases and difficulty of diagnosis and treatment. Consensus treatment has not yet been established. Even with treatment, these pathological changes result in a high mortality rate of over 90%. In China, the first case of PAM caused by *N. fowleri* was reported in Henan in 1979, and several cases of encephalitis caused by FLA were subsequently reported nationwide. There were an increasing number of cases reported in the last decade. There are great differences in the description and analysis of clinical data reported by different research reports.

Encephalitis by FLA can cause severe clinical problems. Three kinds of infections, despite low morbidity, have relatively high mortality rates, which is a considerable challenge for early diagnosis and efficient therapy. This article comprehensively analyzes the literature reports in China and the medical records in our hospital. Combining these clinical data, the article aimed to determine the clinical characteristics and differences among PAM, GAE, and BAE in the Chinese population to develop strategies to manage the three FLA encephalitis.

## 2. Methods

This systematic review was performed according to “Preferred Reporting Items for Systematic Reviews and Meta-Analyses” (PRISMA) statement (6).

### 2.1. Search strategy

Two independent reviewers (Chen XT and Zhang Q) conducted the search. The search databases include MEDLINE (PubMed interface), EMBASE, China National Knowledge Infrastructure (CNKI), Wanfang database, and China Biology Medicine disc (CBMdisc) databases. And manually retrieve the hospital records of our hospital. The search time was up to August 30, 2022, with no language restrictions. All discrepancies were resolved by a third investigator (Zhou CQ). The search keywords used were “*N. fowleri*,” “*Acanthamoeba* spp.,” “*B. mandrillaris*,” “Primary Amoebic Meningoencephalitis,” “Granulomatous Amoebic Encephalitis,” and “Balamuthia Amoebic Encephalitis.”

### 2.2. Inclusion/exclusion criteria

Case reports and case series reporting PAM, GAE, and BAE were included. These studies met the following inclusion criteria: (1) PAM, GAE, and BAE patients were confirmed and diagnosed. (2) Containing raw data for clinical symptoms, diagnosis, treatment, and outcome of the patient was addressed. (3) Region is limited to China.

Studies without enough data, review articles, modeling study, commentary, correspondence, editorial, guidelines, and news were excluded. All potentially relevant articles were then screened for eligibility. Two reviewers (Chen XT and Zhang Q) independently screened the records by title, abstract, and full texts to exclude those not related to the current study. And any disagreements were discussed and resolved by a third investigator (Zhou CQ).

### 2.3. Date collection

The extracted data included the first author's name; the year and place of origin; age and sex of the patient; diagnosis methods; data on clinical, radiological, and laboratory findings; therapy, and the patient outcome. And the proportion of each data in the total number of relevant cases was calculated and analyzed. Data extraction was performed by two reviewers (Wen SY and Chen FF) and any discrepancies were resolved by a third reviewer (Zhou CQ).

### 2.4. Statistical analyses

Statistical analyses were performed using SPSS version 26.0. After testing for normality and homoscedastity, the quantitative data were expressed as median (range), and statistical significance was determined by Kruskal–Wallis one-way analysis of variance followed by Kruskal–Wallis Multiple-Comparison *Z*-Value Test (Dunn's Test). Qualitative data were expressed as *n* (%) and statistical methods were adopted by the chi-square test (Fisher's exact test). Statistical significance was established at two-tailed 0.05 level (*P* < 0.05).

### 2.5. Quality assessment

We used the case reports/case series appraisal checklist supplied by the Joanna Briggs Institute (JBI) to evaluate the quality of the studies. Two reviewers (Wen SY and Chen FF) assessed the quality of each included study independently. Any disagreements were resolved by a third reviewer (Zhou CQ).

## 3. Results

### 3.1. Study selection and general characteristics

The results of various studies including participants' clinical manifestations, diagnosis, treatment, and outcome are reported in Table 1. In the systematic review, after excluding possible duplicate cases, a total of 48 patients (37 males, 11 females) were collected, 1 patient from the medical records of our hospital, and 47 patients from 31 different studies (7–37) (Supplementary Figure S1). The population of Lei' case series (37) accounted for a part of the BAE patients in the review. But individual patient partial level data were not available for this study.

**Table 1:** The clinical characteristics of published reports of three free-living amoebic encephalitis.

**Month and year**	**Region**	**Age**	**Sex**	**Main symptoms at onset**	**Means of diagnosis**	**Medical treatment**	**Causative agent**	**Duration**	**Outcome**
December, 1978^a^	Henan	17	M	Headache, fever, vomiting	Postmortem	Nonspecific	*N. fowleri*	10 days	Died
May, 1990^a^	Zhejiang	21	M	Headache, fever, vomiting	CSF Microscopy after death	Nonspecific	*N. fowleri*	6 days	Died
October, 1991	Hong Kong	38	M	Auditory hallucinations, impaired attention	Amoeba was observed in an abscess biopsy	Amphotericin B + Rifampicin + Chloramphenicol + Nonspecific + Operation	*N. fowleri*	3 months	Moderate disability
August, 2001^a^	Henan	33	M	Headache, fever, vomiting	CSF Microscopy after death	Nonspecific	*N. fowleri*	7 days	Died
2012	Taiwan	75	M	Headache, fever	CSF Microscopy, PCR, DNA sequencing	Amphotericin B	*N. fowleri*	25 days	Died
August, 2016	Zhejiang	42	M	Headache, fever, myalgia	CSF Microscopy, NGS, PCR	Amphotericin B + Azoles (Fluconazole) + Nonspecific	*N. fowleri*	14 days	Died
September, 2020^a^	Fujian	47	M	Fever, confusion	CSF Microscopy, NGS	Amphotericin B + Meropenem + Vancomycin + Nonspecific	*N. fowleri*	64 days	Died
June, 2020	Guangxi	8	M	Headache, fever, vomiting	NGS, PCR, Sanger sequencing after death	Meropenem + Vancomycin + Nonspecific	*N. fowleri*	24 days	Died
August, 2020	Hunan	9	M	Headache, fever, vomiting	CSF Microscopy, NGS, PCR, Sanger sequencing after death	Vancomycin + Nonspecific	*N. fowleri*	4 days	Died
August, 2021^b^	Chongqing	31	M	Headache, fever, disturbance of consciousness	NGS	Amphotericin B + Rifampicin + Trimethoprim-Sulfamethoxazole + Meropenem + Azoles (Metronidazole) + Nonspecific	*N. fowleri*	7 days	Died
September, 2021^a^	Shandong	55	M	Headache, fever, fatigue	CSF microscopy, NGS	Amphotericin B + Rifampicin + Nonspecific	*N. fowleri*	5 days	Died
December, 1972^a^	Jilin	7	M	Hand pain, numbness in fingers	CSF microscopy, postmortem	Nonspecific	*Acanthamoeba rhysodes*	4 years	Died
May, 1983^a^	Beijing	25	F	Headache, nausea, vomiting	Postoperative biopsy	Operation	*Acanthamoeba* spp.	27 days	Died
July, 1987^a^	Beijing	13	M	Fever, headache	Postmortem	Nonspecific	*Acanthamoeba* spp.	27 days	Died
December, 1997^a^	Xinjiang	25	M	Blurred vision	Postmortem	Nonspecific	*Acanthamoeba* spp.	4.5 months	Died
April, 1999^a^	Hunan	25	M	Blurred vision	Postmortem	Nonspecific	*Acanthamoeba* spp.	5 months	Died
2006^a^	Hebei	29	M	Headache, dizziness	Postmortem	Nonspecific + Operation	*Acanthamoeba* spp.	3 months	Died
2007^a^	Guangxi	46	M	Headache, fatigue, seizures	Postoperative biopsy	Nonspecific + Operation	*Acanthamoeba* spp.	1 years	Survived
2009	Taiwan	63	M	Fatigue, difficulty defecation	CSF Microscopy, PCR	Amphotericin B + Rifampicin + Nonspecific	*Acanthamoeba culbertsoni*	78 days	Survived
February, 2021^a^	Fujian	48	F	Confusion, fever, seizures	NGS, Postoperative biopsy	Meropenem + Vancomycin + Azoles (Metronidazole + Voriconazole) + Linezolid + Caspofungin + Tigecycline + Nonspecific	*Acanthamoeba culbertsoni*	23 days	Died
April, 2021^a^	Tianjin	57	M	Headache	NGS	Trimethoprim-Sulfamethoxazole + Azoles (Metronidazole) + Nonspecific	*Acanthamoeba culbertsoni*	1 months	Survived
November, 2018	Jiangxi	9	F	Anorexia, vomiting, fever	NGS	Rifampicin + Azoles (Metronidazole) + Nonspecific	*B. mandrillaris*	24 days	Died
2018	Shanghai	7	M	Skin lesions, gait instability	Skin tissues PCR after death	Nonspecific	*B. mandrillaris*	1 months	Died
2019	Fujian	13	F	Skin lesions, dizziness, vomiting	NGS	Trimethoprim-Sulfamethoxazole + Macrolides (Azithromycin) + Azoles (Albendazole/ Fluconazole) + Amphotericin B + Flucytosine + Nonspecific	*B. mandrillaris*	5 months	Died
2019	Gansu	2	M	Fever	NGS	Meropenem + Nonspecific	*B. mandrillaris*	2 months	Died
May, 2019	Guizhou	15	M	Skin lesions, fever	NGS, skin tissues PCR and Microscopy	Amphotericin B + Macrolides (Azithromycin) + Trimethoprim-Sulfamethoxazole + Azoles (Fluconazole) + Flucytosine + Nonspecific	*B. mandrillaris*	15 days	Died
2020	Guangxi	54	M	Numbness and weakness of left limb	NGS	Amphotericin B + Macrolides (Azithromycin) + Trimethoprim-Sulfamethoxazole + Azoles (Fluconazole) + Flucytosine + Nonspecific + Operation	*B. mandrillaris*	7 months	Survived
2020^a^	Hebei	69	M	Headache, fever	NGS	Amphotericin B + Meropenem + Nonspecific	*B. mandrillaris*	54 days	Died
2021^a^	Beijing	6	M	Headache, vomiting	NGS, Postoperative biopsy	Macrolides (Azithromycin) + Trimethoprim-Sulfamethoxazole + Azoles (Fluconazole) + Flucytosine + Nonspecific + Operation	*B. mandrillaris*	4 months	Died
September, 2021^a^	Hubei	65	F	Headache, fever	NGS	Macrolides (Azithromycin) + Trimethoprim-Sulfamethoxazole + Azoles (Fluconazole + Ornidazole) + Flucytosine + Nonspecific	*B. mandrillaris*	21 days	Died
August, 2021	Guangdong	54	M	Skin lesions, headache, dizzness	NGS	Meropenem + Vancomycin + Azoles (Voriconazole + Albendazole + Metronidazole + Fluconazole)	*B. mandrillaris*	16 days	Died
2022	Zhejiang	37	F	Skin lesions, dizziness	NGS, skin tissues NGS	Amphotericin B + Macrolides (Clarithromycin) + Trimethoprim-Sulfamethoxazole + Azoles (Fluconazole) + Pentamidine + Flucytosine + Nonspecific	*B. mandrillaris*	34 days	Died
2020 (16 patients)	NR	13 (4, 74)	11M, 5F	Skin lesions	Skin tissues PCR, 8 were verified by imaging studies and 8 were verified on the basis of clinical symptoms	Lincomycin + Interferon-γ + Operation (1 Survived)	*B. mandrillaris*	5 days−6 months	15 Died and 1 Survived

Nonspecific treatments include general measures to reduce intracranial pressure and inflammation (mannitol, decompressive craniectomy, and corticosteroids) and treatments for differential diagnosis (cephalosporins for bacterial meningitis). In the case of concomitant medications, the treatments are calculated independently. ^a^Suspected cases documented in Chinese journals. ^b^Not reported. M, male; F, female.

**Figure 1:**
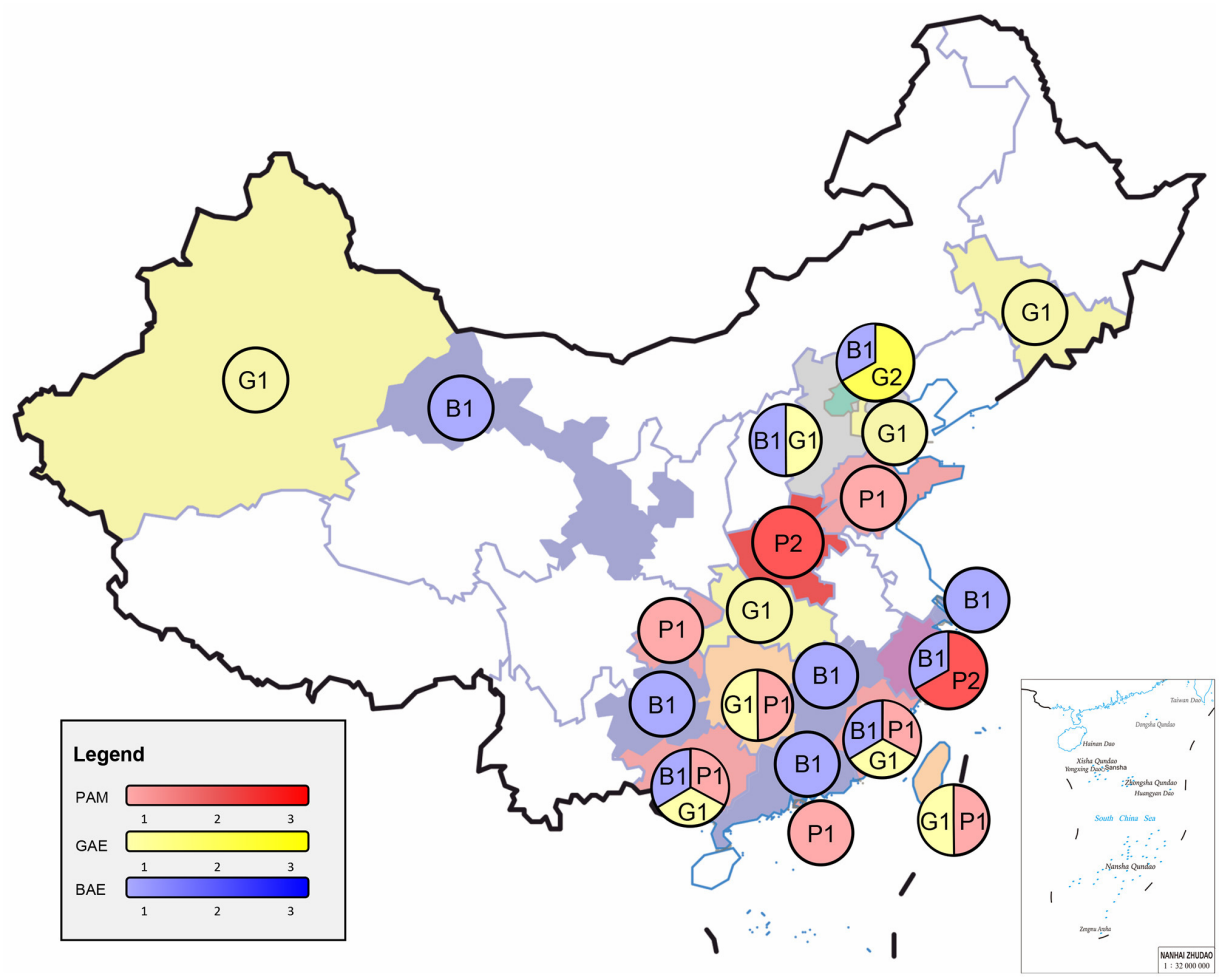
Epidemiology and clinical cases of three free-living amoebae infections in China. PAM, primary amoebic meningoencephalitis; GAE, granulomatous amoebic encephalitis; BAE, balamuthia amoebic encephalitis.

The median age was ~21.0 (2–75) years old. PAM was 33.0 (8–75) years old, and GAE was 27.0 (7-63) years old, and BAE was 13.0 (2-74) years old. A total of 21 patients were under 18 years old, including 3 patients in PAM, 2 patients in GAE, and 16 patients in BAE. Four cases were over 60 years old, including 1 case in PAM, 1 case in GAE, and 2 cases in BAE. More cases are in the South (Figure 1). Data on the season of onset are incomplete, but most patients with PAM present in the summer. All three FLA encephalitis exposure to amoeba-contaminated water or soil sources was possible prior to the onset of symptoms, particularly in PAM (63.6%). Four patients with PAM (including the case in our hospital) swam or bathed in public swimming pools or ponds before the onset of symptoms (7, 14, 15), two patients liked hot springs (9, 11), and one patient attended a Water-Splashing festival and was splashed by lake water (12). The median incubation period for cases with PAM was 4 (1–6) days. Two GAE patients fell into a gully and aspirated muddy water (23, 24). One BAE patient constantly swam in the local ponds (30). A total of 11 BAE patients had a clear history of trauma. Besides, one GAE patient combined with AIDS (25) and one BAE patient combined with breast cancer (36).

### 3.2. Clinical manifestations

The three FLA encephalitis clinical symptoms and signs are in Table 2. The onset of PAM is mostly acute or subacute, and the clinical symptoms are acute and fulminant hemorrhagic meningoencephalitis. Approximately 81.8% of patients initially present with headache, and fever. Most patients with GAE have an insidious onset and a chronic course. GAE usually begins with headache and fever, but also has fatigue and paresthesia as the first symptoms. The headache and vomiting were moderate or mild, and the symptoms of intracranial hypertension were not obvious. Two patients (19, 20) with GAE had ocular symptoms as the onset symptom. The clinical symptoms of BAE are similar to those of GAE. In BAE, 21 (77.8%) patients had skin lesions before the onset of symptoms. In 18 of these patients, the lesions occurred on the face, especially around the nose (7 patients); two patients had skin lesions in the knee; one patient had skin lesions in the left thigh.

**Table 2:** The initial or main clinical symptoms and signs of 48 clinical cases.

**Symptoms and signs**	**No. of case (% of cases)**		
	**PAM (*****n*** = **11)**	**GAE (*****n*** = **10)**	**BAE** **(*****n*** = **27)**
Headache	9 (81.8)	4 (40.0)	16 (59.3)
Fever	9 (81.8)	4 (40.0)	12 (44.4)
Vomiting	5 (45.5)	2 (20.0)	8 (29.6)
Hemiplegia	4 (36.4)	4 (40.0)	4 (14.8)
Visual impairment	3 (27.3)	3 (30.0)	5 (18.5)
Seizures	6 (54.5)	2 (20.0)	3 (11.1)
Mental disorder	2 (18.2)	2 (20.0)	9 (33.3)
Myalgic	2 (18.2)	0	0
Myoclonus	1 (9.1)	1 (10.0)	0
Fatigue	2 (18.2)	1 (10.0)	4 (14.8)
Poor appetite	1 (9.1)	0	1 (3.7)
Dizziness	0	1 (10.0)	4 (14.8)
Unable to walk	0	0	4 (14.8)

### 3.3. Auxiliary examination

#### 3.3.1. Laboratory examination

Complete blood counts are usually nonspecific, and leukocytosis is most common. The cerebrospinal fluid (CSF) findings were abnormal in most patients (Table 3). Reference values were obtained from reference (38). For PAM, CSF pressure increased, significantly increased white blood cell number (median 560 × 10^6^/L, [range 50, 9820]), mainly neutrophils (median 72%, [range 5, 91]), protein increased, glucose content normal or decreased, and chloride content decreased. For GAE CSF pressure is normal or increased, the white blood cell count is slightly increased (median 48 × 10^6^/L, [range 9, 119]), the protein is increased, the glucose content is normal or increased, and the chloride content is normal or slightly decreased. For BAE CSF pressure increased, the number of white blood cells increased significantly (median 315 × 10^6^/L, [range 66, 620]), mainly lymphocytes (median 80.0%, [range 55, 97]), protein increased, the glucose content is normal or increased, and chloride content decreased.

**Table 3:** Initial laboratory findings for three free-living amoebae infections in China.

**Test (reference value)**	**PAM (*****n*** = **11)**		**GAE (*****n*** = **10)**		**BAE (*****n*** = **11)**		***P*-value**
	**Median (range)**	***n***	**Median (range)**	***n***	**Median (range)**	***n***	
**Complete blood count**							
WBC (4.0–10.0) × 10^9^/L	12.7 × 10^9^ (4.6, 41.6)	10	8.6 × 10^9^ (1.0, 10.9)	6	5.5 × 10^9^ (4.3, 10.8)	5	**0.044**
% Neutrophils (50–70%)	89 (76, 94.6)	11	77 (62.8, 83.7)	4	71 (31.7, 90)	4	**0.027**
% Lymphocytes (20–40%)	9 (3,24)	6	19 (13.9, 23)	2	19 (5.1, 59)	3	0.441
**Cerebrospinal fluid**							
Opening pressure (80–180 mm H_2_O)	300 (240, 475)	9	200 (30, 250)^a^	8	250 (220, 330)^b^	7	**0.002**
RBCs (0 cells/μL)	200 (15, 300)	3	101 (100, 102)	2	200 (30, 216)	3	0.799
WBCs (0~8) × 10^6^/L	560 × 10^6^ (50, 9820)	9	48 × 10^6^ (9, 119)^a^	6	315 × 10^6^ (66, 620)	8	**0.006**
% Neutrophils	72 (5, 91)	10	20 (20, 68)	3	22 (3, 90)	5	0.096
% Lymphocytes (70%)	29 (5,30)	4	30 (15, 70)	3	80 (55, 97)^a^	5	**0.022**
Protein (0.20–0.40 g/L)	2.83 (0.50, 26.06)	11	0.95 (0.51, 1.60) ^a^	8	1.49 (0.51, 4.71)	10	**0.040**
Glucose (2.50–4.40 mmol/L)	0.50 (0.01, 3.40)	9	3.80 (1.78, 6.20)^a^	8	1.92 (1.42, 3.96)	10	**0.007**
Chloride (120–130 mmol/L)	107 (101, 120)	4	120 (117, 122.2)	6	117 (106.4, 129.6)	7	0.115

P-values were derived from Kruskal–Wallis (K–W test) test. ^a^Compared with the PAM patient group, P < 0.05; ^b^Compared with the GAE patient group, P < 0.05.

Values in bold indicate statistically significant results.

#### 3.3.2. Imaging examination

**Figure 2:**
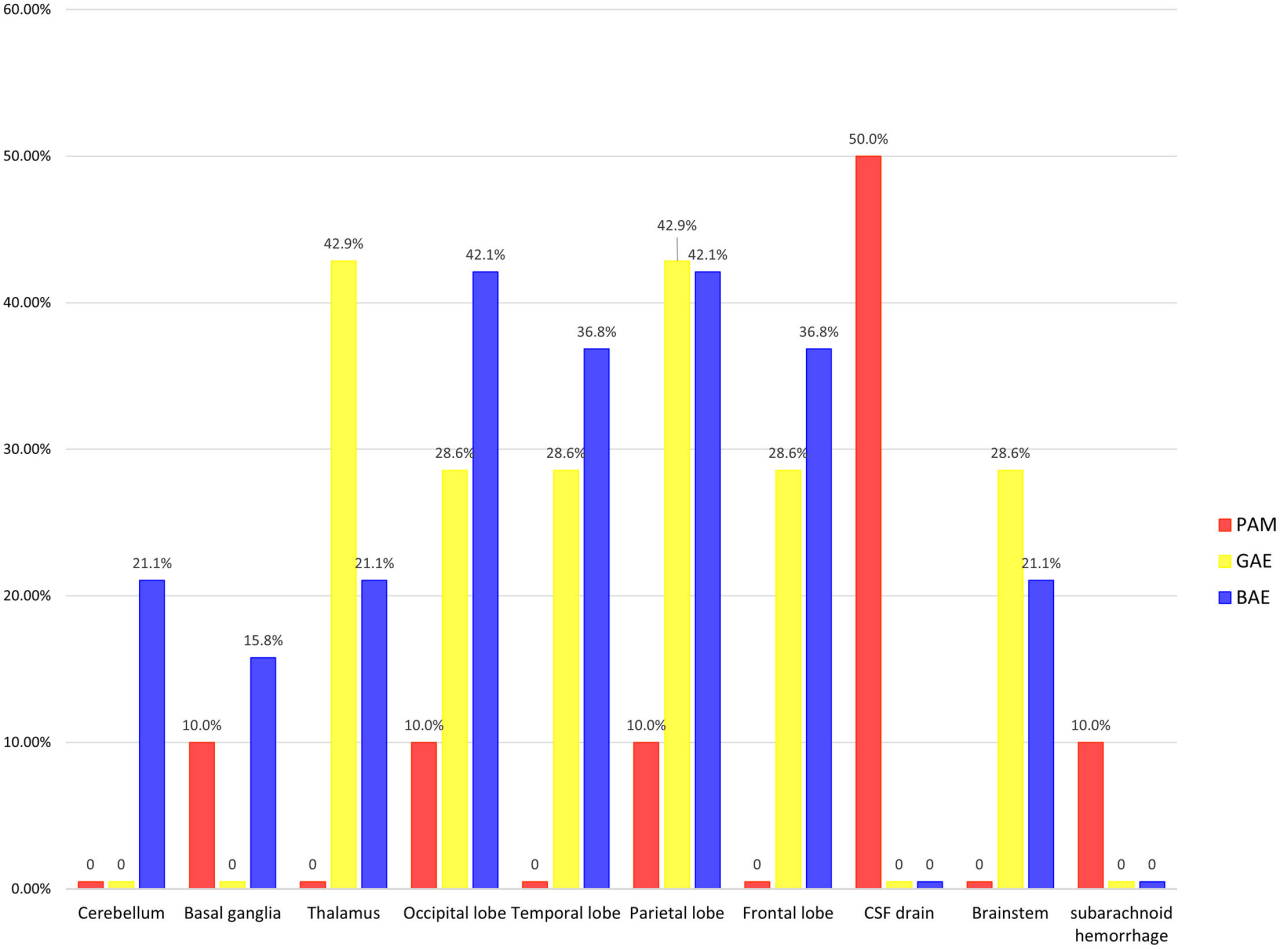
Sites of infection of three free-living amoebae infections in China.

Imaging examinations included computed tomographic (CT) scanning (32 cases), magnetic resonance imaging (MRI) (30 cases), and a combination of CT scanning and MRI (22 cases). We selected the first imaging at the first admission for analysis. Moreover, when two imaging modalities were used simultaneously during the first admission, MRI was considered to be superior to CT scanning in terms of demonstrating focal lesions that were evolving over time. Therefore, we prioritized MRI images and descriptions over CT images and descriptions (Figure 2). A total of 10 PAM patients, 7 GAE patients and 19 BAE patients had definite imaging examinations.

For PAM, it was most frequently observed that the CSF drainage system (with hydrocephalus) was favored in 5 (50.0%) cases. Notably, one case of PAM and GAE showed normal neuroimaging findings. For GAE, 3 (42.9%) cases were reported to have lesions in the thalamus and 3 (42.9%) in the parietal lobe. For BAE, 8 (42.1%) cases were reported to have lesions in the occipital lobe and 8 (42.1%) in the parietal lobe.

### 3.4. Diagnostic methods

For PAM, 6 (54.5%) patients were diagnosed premortem. Among the premortem cases, CSF microscopy was used successfully in 4 (66.7%) patients and next generation sequencing (NGS) was used in 4 (66.7%) of cases. In one case (12), the group assembled a 1.6-kb consensus sequence of the 18S ribosomal RNA of *N. fowleri* and uploaded it to the GenBank (GenBank accession no.KY062165). For GAE, 5 (50.0%) patients were diagnosed premortem. Among the premortem cases, postoperative biopsy was used in 3 (60.0%) of cases, and CSF NGS was used in 2 (40.0%) of cases and CSF microscopy were used successfully in 1 (20.0%) of cases. Of the 10 patients with GAE, two patients were confirmed by CSF NGS to be *Acanthamoeba culbertsoni*, and one patient was confirmed to have strong homology with *Acanthamoeba culbertsoni* by CSF Polymerase chain reaction (PCR). One patient was confirmed by immunofluorescence analysis and the pathogen was *Acanthamoeba rhysodes*. The pathogens of the remaining 6 GAE patients were confirmed by autopsy or postoperative pathology to be *Acanthamoeba* spp. For BAE, ~26 (96.3%) cases were identified premortem. Among the premortem cases, NGS was used positively in 10 (38.5%) of cases. Skin tissue PCR was used successfully in 18 (69.2%) of cases (Table 1).

### 3.5. Treatments and outcomes

We summarize all the therapeutic regiment, including survivors' (Table 1). Among these, the most frequently used agents included amphotericin B (12 cases), azoles (12 cases), and rifampicin (5 cases) in regimens ranging from one to four total antimicrobials. In addition, surgical treatment and non-specific treatments such as general measures to reduce intracranial pressure and inflammation are also included. For survived patients, therapeutic regiment in PAM included amphotericin B, rifampicin, and chloramphenicol. Furthermore, decompressive occipital lobectomy, insertion of a ventricular catheter, and removal of the bone flap were also perfomed actively. Among GAE survivors, one received surgery, one received amphotericin B and rifampicin, and one received trimethoprim-sulfamethoxazole, metronidazole. Among BAE survivors, one received amphotericin B, azithromycin, trimethoprim-Sulfamethoxazole, azoles, flucytosine, and operation. Another survivor accepted lincomycin, interferon-γ for skin lesion, and operation for brain focal infection.

Among the 48 clinical cases that we gathered from China, only 6 survived (1 for PAM, 3 for GAE, and 2 for BAE). The only PAM survivor was dead 13 months after discharge with left hemiplegia, conjugate gaze paralysis, and hemisensory neglect (9). One survivor with GAE has returned to normal work and life, and has no encephalitis symptoms after 1 year of follow-up (25). One survivor with BAE was discharged from the local hospital and showed good recovery with an activity of daily living (ADL) scores of 95 (31).

### 3.6. Risk of bias assessment

The results of the critical appraisal (JBI checklist) of included studies are summarized in Supplementary Table S1. Overall, the quality assessment of the eligible studies revealed that all the recommended elements were fulfilled basically. Thus, these were considered low risk of bias.

## 4. Discussion

To our knowledge, this article is the largest clinical case series of patients from China with three pathogenic FLA encephalitis, including PAM, GAE, and BAE. There are obvious differences and similarities between three FLA encephalitis. We analyzed the exposure location, clinical symptoms, diagnosis, treatment, and outcomes, and hope it will be helpful to early differential diagnosis of different meningoencephalitis and initiate amoebic-specific therapy more rapidly.

In our study, BAE has almost 3 times more cases than PAM or GAE, but this may not indicate a greater susceptibility to *B. mandrillaris*. In general, the sample size on free-living amoebic encephalitis is limited in China. BAE has a slower disease course than PAM. And because of the manifestation of skin lesions before the appearance of encephalitis symptoms in most of BAM patients, BAE may be more likely to be identified and reported. Besides, in the last 5 years, BAE has been reported more frequently than PAM and GAE, and medical personnel are more aware of BAE. There is also a case series on *B. mandrillaris* infection (including 16 patients with BAE) in China (37). The above may be the reason for the number of cases of BAE was higher than that of other PAM and GAE.

The susceptible population of three FLA encephalitis are mostly healthy young people and children. Patients were predominantly male, especially PAM. And BAE is more likely to occur in younger age groups. The reason for this pattern of sex and age distribution is unclear. The increased number of infections in young males may be related to differences in exposure to outdoor activities. Some behaviors, hobbies, or works could be putting these people at a higher risk. Such as engage in agricultural activities, or dive, swim, jump into water in rivers or ponds, or soak in a hot spring, increasing the risk of skin breakdown or water forcing its way into the nasal cavity. These activities have been documented in some patients. However, these activities also do not fully explain the increased risk of infection. These are similar to the susceptible populations in the United States (39, 40), India (41), Thailand (42), and Peru (43). But our group seems to find that the median age of PAM in China is higher than in the rest of the globe, for example, the United States (39). This may be due to the fact that some high-risk behaviors, hobbies, or works described above are more common among adults in China. Among the GAE and BAE patients reported in the United States (44) and Peru (43, 45, 46), it is more likely to occur in immunocompromised patients. Similarly, China did show similar epidemiologic patterns. Immunocompromised youngers due to tumors or chronic diseases (e.g., breast cancer, AIDS, long-term glucocorticoids use, and skin lesions) may increase the risk of contracting GAE or BAE or accelerating the course of GAE or BAE. And immunocompromised patients have relatively poorer outcomes in later treatment. The southern regions of China reported cases more frequently. Many FLA cases also been reported in tropical regions of the world, which may be associated with the dry and hot climate environment. Besides, southern China generally has a higher economic and medical level than northern China, with a greater ability to identify and report cases. A variety of factors may have contributed to this regional trend. And there were an increasing number of cases reported in southern China over time. It is also likely due to improvements in awareness and diagnostic capacity. Broader case reporting is needed to validate this epidemiologic pattern. Besides, we also found that PAM cases with data on a clear season of onset were mostly in the warm, hot summer months, which is consistent with the thermophilic nature of *N. fowleri*. All three FLA encephalitis exposure to amoeba-contaminated water or soil sources was possible prior to the onset of symptoms. But unfortunately, in only one case (23) did the research group collect both a sample of the patient's CSF and an aqueous sample of the patient's inhaled muddy water. The results indicated that the *Acanthamoeba* spp. indeed, existed in the rice field.

The onset of PAM is mostly acute or subacute. The median incubation period for PAM cases in China was 4 (1–6) days, similar to other countries. We found that time from onset of symptoms to death of PAM patients in China take longer than the rest of the world (47). It has been reported that different geographic distribution of each genotype isolated from environmental and clinical specimens (48). But there is no conclusive evidence to prove different types of *N. fowleri* have virulence differences. Besides, we found that although Chinese PAM patients had a long disease duration, their disease progressed quickly. Most of the several patients with longer disease duration had motile amoeba or trophozoite-like organisms found in the CSF and were anti-amoebic treated accordingly. However, by the time the diagnosis was clear, the patients were in a coma and anti-amoebic treatment did not save their lives. Timely anti-amoebic treatment may prolong the disease course. Most patients with GAE and BAE have an insidious onset and a chronic course.

The clinical manifestations of the three encephalitis are similar, but there are some significant differences. Neurological symptoms of PAM present as acute and fulminant hemorrhagic meningoencephalitis. GAE and BAE have a slower disease progression than PAM. Two patients (19, 20) with GAE had ocular symptoms as the onset symptom, which is noteworthy. The genus *Acanthamoeba* is currently classified into 23 genotypes (T1–T23), and of these some are known to be capable of causing GAE mainly in immunocompromised patients while other genotypes cause *Acanthamoeba* keratitis (AK) mainly in otherwise healthy patients. There are partial genotypes known to cause both AK and GAE (T2, T4, T5, T10, and T12) (49). Whether ocular symptoms can be indicative of GAE is a question that needs to be further explored in future studies. In BAE, 77.8% of patients initially had skin lesions, the most common lesion seen is a painless fuchsia plaque. And develop encephalitis several months or years later. The most common location is on the central face, over the nose, or on one extremity, which is similar to cases reported in Peru (43, 45, 46). The patients may develop encephalitis several months or years after skin lesions. However, cases from the US often progress directly to encephalitis without skin lesions (40). It is not known why some patients initially develop skin lesions while others develop encephalitis directly. Different genotypic differences in geographically distributed *B. mandrillaris* may lead to difference in pathobiology. But *B. mandrillaris* is the only pathogenic species without genotypic classification. It has been reported the variable length of the rps3 type II intron (region of the mitochondrial genome) between the different strains of *B. mandrillaris* suggesting that it may be promising for the development of molecular genotyping (49). More *B. mandrillaris* from different geographical locations need to be sequenced for molecular genotyping. Besides, we hypothesized that it also may be related to the invasion pathways. A total of 11 patients had a clear history of trauma. China has a large rural population with a high risk of skin trauma and skin infection compared to the urban population.

The precise diagnosis of the disease and the causal agent promptly is the critical step to deliver the appropriate treatment. It is challenging to diagnose and differentiate PAM, GAE, and BAE by the laboratory or imaging examination, and the results were similar to those observed in the United States (44). But the specificity of neuroimaging for the diagnosis has yet to be evaluated. PAM in neuroimaging mostly had only one infected lesion with diffuse cerebral edema and signs of increased intracranial pressure. Besides, one case of PAM showed normal neuroimaging findings, but the patient had already been experiencing acute symptoms such as headache, fever, and confusion for 2 days. Normal imaging findings were also observed early in a patient with GAE who presented with only headache symptoms. This false-negative result was also seen in a patient in the United States (50), which is worrisome. Patients may not receive early intervention if medical professionals are not aware of the disease. Neuroimaging of GAE and BAE typically shows multiple, well-defined, focal, ring-enhancing, space-occupying lesions. Brain lesions in the thalamus and parietal lobe were much more frequent for GAE. The frontal lobe, parietal lobe, temporal lobe, and occipital lobe were affected most in cases of BAE.

Moreover, among patients diagnosed premortem, 4 of PAM, 2 of GAE, and 10 of BAE patients were diagnosed by NGS. NGS constitutes a rapid and accurate method for pathogen identification, particularly in diagnosing unexplained diseases. Its turnover time is < 48 h, and is less affected by previous antibiotic exposure (51). And the samples for NGS analysis are generally flexible, including CSF, blood, tissue, etc. This unbiased NGS provides an operable diagnosis for rare pathogenic infections, which is helpful for clinicians to timely diagnose and find targeted and effective treatment. Although the cost of NGS testing is significantly lower compared to Sanger sequencing, it is still very expensive compared to other traditional laboratory tests. Currently, a single NGS test can cost up to six hundred dollars. The high cost is one of the factors that limit the widespread use of NGS in clinical settings. But with the further maturation of the technology, NGS should be further improved in the future in terms of cost reduction, diagnostic standard improvement, quality control improvement, etc. Therefore, we propose that the NGS method should be applied more widely in the clinical practices to help physicians make diagnosis. Suspicion of FLA encephalitis should be raised when the presence of motile amoeba is found in CSF wet-mount smear or trophozoite-like organisms is found in CSF by Giemsa stains. *N. fowleri* was found in the CSF more often than the other two types of amoebae, most likely because of its characteristic motile flagellated form. It is worth noting that negative CSF samples findings cannot exclude the possibility of infection in suspected patients. Inadequate atypical morphological features are difficult to distinguish different amoeba. If an amoeba is detected in CSF, or there are associated risk factors, such as the history of freshwater exposure, skin lesions, or ineffective antibiotic therapy, confirmatory testing by immunohistochemical staining, PCR, or NGS are required for the diagnosis of FLA encephalitis. For BAE, if the patients present first with a skin lesion, they may expect to be more easily diagnosed by skin biopsy, immunohistochemical staining, PCR or NGS. Therefore, it is important to correctly identify the cutaneous manifestation of the disease.

Life expectancy very poor, with only 6 successful treatment cases reported in China. No single agent can be proposed as the ideal therapy by itself. Amphotericin B is an antifungal drug, and has been used in the treatment of PAM since 1970. Although amphotericin B is still considered the drug of choice for treating PAM, it has limited efficacy and some side effects, such as anemia, fever, nausea, and dose-related nephrotoxicity. It has been reported that amphotericin B was administered to most confirmed PAM patients, but only 7 patients survived and also received other treatments (47). The Centers for Disease Control and Prevention (CDC) in the United States based on case reports or *in vitro* studies recommends the standard treatment for PAM, which is a combinational therapeutic cocktail that includes amphotericin B, an azole (ketoconazole, fluconazole, miconazole), azithromycin, rifampin, and miltefosine (52, 53). However, treatment may be successful only at early stages. The onset of PAM is urgent and progresses rapidly in a short period of time, and there is often not receiving targeted and effective treatment in time. The only surviving PAM patient in China had a less acute presentation. And he had received amphotericin B, in combination with rifampicin, chloramphenicol, and other non-specific treatments. Including decompressive occipital lobectomy, insertion of a ventricular catheter and removal of the bone flap to reduce intracranial pressure actively. This may be the key to the survival of the patient. Whether chloramphenicol has any anti-amoebic effect remains unproven. Miltefosine is originally developed as an anticancer drug, and currently used as an effective antileishmanial therapy. Because it was used to treat the two survivors in the United States in 2013 (39). Recent reports showed good *in-vitro* activity of miltefosine against *B. mandrillaris, Acanthamoeba* spp., and *N. fowleri* species (54–57). But it's not yet available in China.

The treatment of GAE usually involves combination therapy also. The available drugs include liposomal amphotericin B, rifampin, azoles (voriconazole, itraconazole, fluconazole, ketoconazole), caspofungin, sulfadiazine, pyrimethamine, cotrimoxazole (trimethoprim–sulfamethoxazole), meropenem, linezolid and moxifloxacin, pentamidine isethionate and miltefosine (3, 56, 58). The 2007 survivor was aggressively managed for his elevated intracranial pressure, including anti-infective therapy and lesion excision surgery. The 2009 survivor was given amphotericin B, Rifampicin, and other nonspecific. The 2021 survivor was given cotrimoxazole and metronidazole. However, one GAE patient in 2021 was already in critical condition when she received targeted medication after the diagnosis was clear. And this patient's disease progressed extremely rapidly. He passed away despite receiving aggressive treatment with most of the recommended medications. These cases proved that treatment effects may be related to the severity and duration of the disease, and early diagnosis.

The treatment of one BAE survivor in China consisted of amphotericin B, macrolides (azithromycin), sulfonamides, azoles (fluconazole), and flucytosine. Because there are so few surviving cases, this treatment option requires further research. We observed one patient who was treated with essentially a similar regimen, but unfortunately passed away. This may be related to this patient progressing very rapidly after the onset of brain symptoms. This drug combination, may still represent the best choice for current treating BAE until new drugs are developed in further. The second survivor was treated with lincomycin and interferon-γ mainly for skin lesion. The brain lesion was detected by imaging before the onset of brain symptoms, and the medicine for encephalitis is not reported. Thereafter, it was surgically excised and cured. Based on a limited number of successful clinical cases and previously identified *in vitro* drug susceptibility, CDC recommends the therapeutic drug for BAE consists of pentamidine, 5-flucytosine, fluconazole, and a macrolide (clarithromycin or azithromycin) with one of the following: sulfadiazine, miltefosine, thioridazine or liposomal amphotericin B (53, 59). Recently, Laurie et al. (60) screened more than 2000 drugs and identified *in vitro* efficacy of quinoline nitroxoline is better than that of other antiamebic medications; it also is safe and well tolerated. A patient in the United States survived after receiving treatment with a regimen that included the repurposed drug nitroxoline (61). We believe that quinoline nitroxoline maybe a promising treatment for *B. mandrillaris* infection.

We suggest clinicians should ideally investigate for less common potential pathogens, in patients with bacterial meningitis who are unresponsive to first-line antibiotic treatments. And treatment strategies usually combine various classes of drugs with different action mechanisms for individualized therapies. In addition to anti-amoebic medications, they are managed with physical procedures such as CSF drainage, hyperosmolar therapy, moderate hyperventilation, and hypothermia. Cerebral hernia is a common cause of death. Therefore, management of high intracranial pressure should be promptly instituted. External ventricular draining is the preferred procedure (62). Diagnostic biopsy is previously applied in three FLA encephalitis. It is now suggested that early surgical resection of intracranial lesions combined with drug therapy may be an important method for the treatment of three FLA encephalitis. In China, one survivor in PAM, one survivor in GAE, and two survivors in BAE were treated by excision of the infected brain tissue. However, due to the limited number of surgical survivors, prognostic factors and effective treatment options need to be further studied.

Some inherent selection bias may limit this article due to differences in medical experience, and the ability to identify and report cases in different provinces and cities. And FLA encephalitis is a rare case, the sample size is small, and the conclusions are limited. Patients present with fever, headache and vomiting, which are very similar to bacterial or viral encephalitis and are very prone to misdiagnosis. Misdiagnosis is more likely to occur when FLA is coinfected with bacteria and viruses. This misdiagnosis can easily result in patients not receiving timely and effective treatment, since FLA is the main responsible for encephalitis. Due to the common misdiagnosis of bacterial meningitis, numerous cases may remain unreported.

## 5. Conclusions

FLA encephalitis is a rare but pathogenic infection that currently lacks specificity in clinical, laboratory, and imaging manifestations, despite advances in clinical understanding, diagnostic methods, and treatment. Increasing awareness of FLA encephalitis could improve the detection and reporting of cases, thereby increasing our knowledge of the disease. Rapid clinical diagnosis relies primarily on the vigilance of clinical physicians and rapid pathogenic detection methods. NGS of the CSF is an unbiased and rapid method, and it can lead to earlier diagnosis and intervention. Therefore, we emphasize the early identification of encephalitis caused by this less common pathogen to enable early diagnosis and subsequent treatment, improving patient outcomes.

## Author contributions

X-TC, QZ, and C-QZ participated in the study concept and design. X-TC, S-YW, and F-FC participated in the acquisition, statistical analysis, and interpretation of the results. X-TC, QZ, S-YW, F-FC, and C-QZ prepared the manuscript. X-TC and C-QZ revised the draft paper for content. All authors contributed to the article and approved the submitted version.
