# Supplementary material for: Pathogenic free-living amoebic encephalitis from 48 cases in China: A systematic review
Source: Front Neurol. 2023 Feb 9;14:1100785. doi: 10.3389/fneur.2023.1100785 (PMC9947844; doi:10.3389/fneur.2023.1100785)
Supplement: Supplementary file 1 [file Table_1.DOCX]

| **Figure S1.** Flowchart of the employed literature search. |
| --- |
| 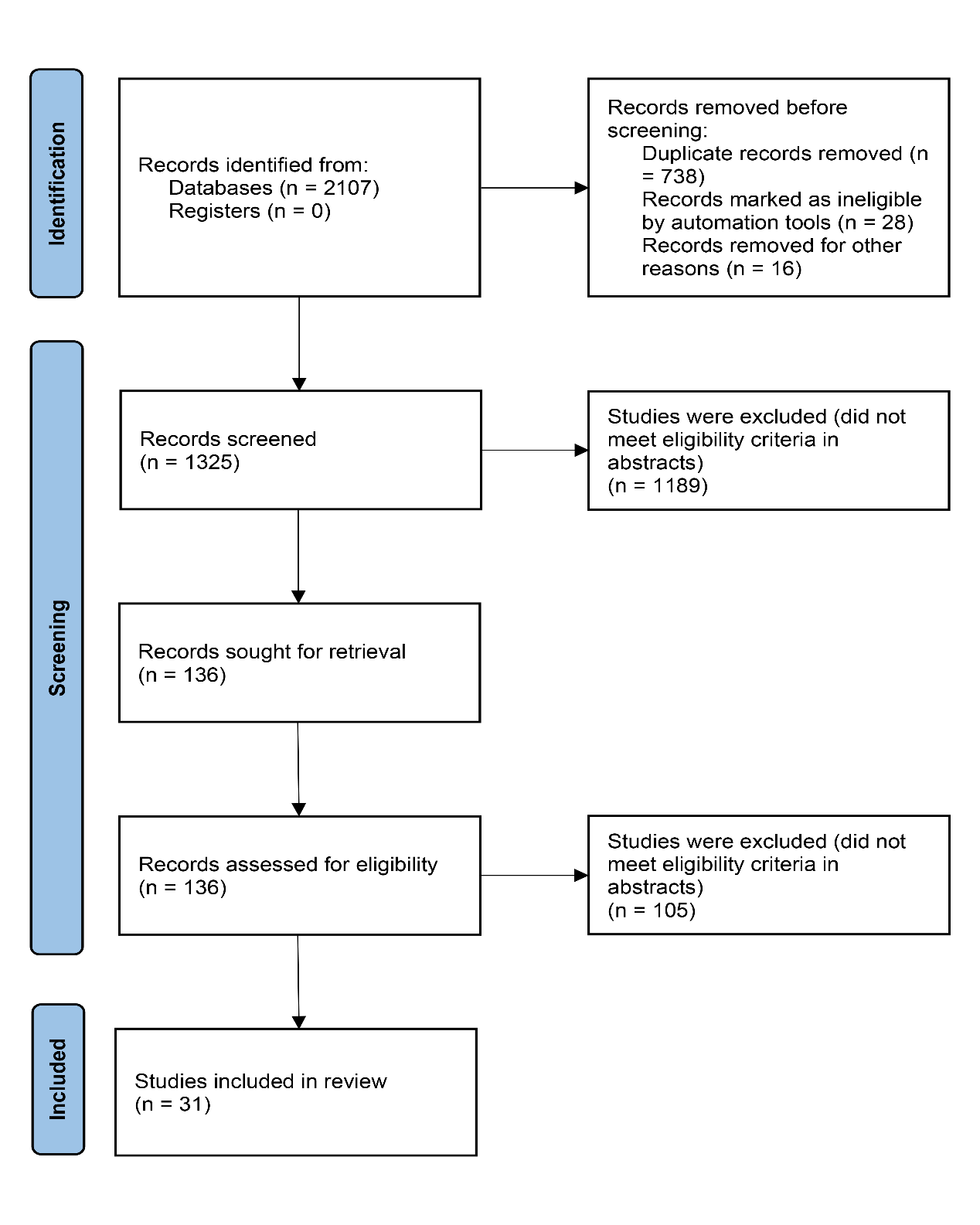 |

| **Table S1.** Quality Assessment of Included Case Reports. | | | | | | | | |
| --- | --- | --- | --- | --- | --- | --- | --- | --- |
| Author, year | Q1 | Q2 | Q3 | Q4 | Q5 | Q6 | Q7 | Q8 |
| Fang et al., 1984^a^ | Y | Y | Y | Y | Y | Y | N | Y |
| Zhao et al., 1991^a^ | Y | Y | Y | Y | Y | Y | N | Y |
| Wang et al., 1993 | Y | Y | Y | Y | Y | Y | N | Y |
| Duan et al., 2002^a^ | Y | Y | Y | Y | Y | Y | N | Y |
| Su et al., 2013 | Y | Y | Y | Y | Y | Y | N | Y |
| Wang et al., 2018 | Y | Y | Y | Y | Y | Y | N | Y |
| Chen et al., 2022^a^ | Y | Y | Y | Y | Y | Y | N | Y |
| Huang et al., 2021 | Y | Y | Y | Y | Y | Y | N | Y |
| Zhou et al., 2020 | Y | Y | Y | Y | Y | Y | N | Y |
| Wu et al., 2021^a^ | Y | Y | Y | Y | Y | Y | N | Y |
| Zhou et al., 1985^a^ | Y | Y | Y | Y | Y | Y | N | Y |
| Lu et al., 1991^a^ | Y | Y | Y | Y | Y | Y | N | Y |
| Shang et al., 1998^a^ | Y | Y | Y | Y | Y | Y | N | Y |
| Chen et al., 1999^a^ | Y | Y | Y | Y | Y | Y | N | Y |
| Zhu et al., 2007^a^ | Y | Y | Y | Y | Y | Y | N | Y |
| Guo et al., 2008^a^ | Y | Y | Y | Y | Y | Y | N | Y |
| Sheng et al., 2009 | Y | Y | Y | Y | Y | Y | N | Y |
| Chen et al., 2021^a^ | Y | Y | Y | Y | Y | Y | N | Y |
| Fan et al, 2022 | Y | Y | Y | Y | Y | Y | N | Y |
| Yi et al., 2020 | Y | Y | Y | Y | Y | Y | N | Y |
| Zhang et al., 2022 | Y | Y | Y | Y | Y | Y | N | Y |
| Wu et al., 2020 | Y | Y | Y | Y | Y | Y | N | Y |
| Yang et al., 2020 | Y | Y | Y | Y | Y | Y | N | Y |
| Ai et al., 2022 | Y | Y | Y | Y | Y | Y | N | Y |
| Peng et al., 2022 | Y | Y | Y | Y | Y | Y | N | Y |
| Yuan et al., 2020^a^ | Y | Y | Y | Y | Y | Y | N | Y |
| Dai et al., 2022^a^ | Y | Y | Y | Y | Y | Y | N | Y |
| Yang et al., 2022^a^ | Y | Y | Y | Y | Y | Y | N | Y |
| Xu et al., 2022 | Y | Y | Y | Y | Y | Y | N | Y |
| Hu et al., 2022 | Y | Y | Y | Y | Y | Y | N | Y |
| Lei et al., 2020 (16 patients) | Y | N | Y | Y | Y | Y | N | Y |
| (1) Were patient’s demographic characteristics clearly described ?; (2) Was the patient’s history clearly described and presented as a timeline?; (3) Was the current clinical condition of the patient on presentation clearly described?; (4) Were diagnostic tests or assessment methods and the results clearly described ?; (5) Was the intervention(s) or treatment procedure(s) clearly described?; (6) Was the post-intervention clinical condition clearly described?; (7) Were adverse events (harms) or unanticipated events identified and described?; (8) Does the case report provide takeaway lessons?N: No; Y: Yes; U: Unclear; N/A: Not Applicable. | | | | | | | | |
